# The effect of vitamin K supplementation on cardiovascular risk factors: a systematic review and meta-analysis

**DOI:** 10.1017/jns.2023.106

**Published:** 2024-01-11

**Authors:** Qiu-Yan Zhao, Qiu Li, Minoo Hasan Rashedi, Mohammadhassan Sohouli, Pejman Rohani, Periyannan Velu

**Affiliations:** 1 Department of Nephrology, the First People's Hospital of Shuangliu District (West China Airport Hospital of Sichuan University), Chengdu, 610041, China; 2 General Practice Ward/International Medical Center Ward, General Practice Medical Center, West China Hospital, Sichuan University/West China School of Nursing, Sichuan University, Chengdu 610041, China; 3 Iran University of Medical Sciences, Tehran, Iran; 4 Student Research Committee, Department of Clinical Nutrition and Dietetics, Faculty of Nutrition and Food Technology, Shahid Beheshti University of Medical Sciences, Tehran, Iran; 5 Pediatric Gastroenterology and Hepatology Research Center, Pediatrics Centre of Excellence, Children's Medical Center, Tehran University of Medical Sciences, Tehran, Iran; 6 Department of Biochemistry and Biotechnology, Faculty of Science, Annamalai University, Chidambaram, Tamil Nadu, India

**Keywords:** Cardiovascular disease, Glucose, Lipid profile, Meta-analysis, Vitamin K

## Abstract

Cardiovascular disease (CVD) is one of the most important diseases which controlling its related risk factors, such as metabolic and inflammatory biomarkers, is necessary because of the increased mortality risk of that. The aim of our meta-analysis is to reveal the general effect of vitamin K supplementation on its related risk factors. Original databases were searched using standard keywords to identify all randomized clinical trials (RCTs) investigating the effects of vitamin K on CVD. Pooled weighted mean difference (WMD) and 95 % confidence intervals (95 % CI) were achieved by random-model effect analysis for the best estimation of outcomes. The statistical heterogeneity was determined using the Cochran's *Q* test and *I*^2^ statistics. Seventeen studies were included in this systematic review and meta-analysis. The pooled findings showed that vitamin K supplementation can reduce homeostatic model assessment insulin resistance (HOMA-IR) (WMD: −0⋅24, 95 % CI: −0⋅49, −0⋅02, *P* = 0⋅047) significantly compared to the placebo group. However, no significant effect was observed on other outcomes. Subgroup analysis showed a significant effect of vitamin K2 supplementation compared to vitamin K1 supplementation on HOMA-IR. However, no significant effect was observed on other variables. Also, subgroup analysis showed no potential effect of vitamin K supplementation on any outcome and omitting any articles did not affect the final results. We demonstrated that supplementation with vitamin K has no effect on anthropometrics indexes, CRP, glucose metabolism, and lipid profile factors except HOMA-IR.

## Introduction

Cardiovascular disease (CVD) is one of the most dangerous groups of non-communicable diseases, which caused the death of 18⋅6 million people in 2019 that 58 % of these deaths were in Asia.^(1,2)^ Metabolic syndrome, as a set of risk factors, is closely related to CVD. Glucose intolerance, hyperinsulinemia, insulin resistance, hypertriglyceridemia, decreased high-density lipoprotein (HDL), and increased small-dense low-density lipoprotein (LDL) are the most important blood changes caused by this syndrome. These changes also cause inflammation in the body, which is characterized by an increase in C-reactive protein (CRP) and other inflammatory biomarkers. Obesity, also characterized by weight gain, body mass index (BMI), waist circumference (WC), and increased central fat, is one of the results of metabolic syndrome, leading to CVD.^(3)^ In addition to metabolic syndrome, high blood pressure (BP), especially systolic blood pressure (SBP), is a major risk factor for CVD and death from the disease; it is responsible for more than half of all deaths from stroke and ischaemia.^(2)^

Ways to treat and control this disease are diet modification, weight regulation, exercise, regulation of lipid profile, and BP, as well as non-smoking and other drugs. Despite recognizing many risk factors and ways to correct them, for reasons such as lack of proper knowledge transfer to patients or unwarranted fear of some patients taking some of their drugs, the disease still remains a threat to human society.^(4)^

The effect of nutrition on CVD has been studied in many studies, both as the effect of a single nutrient and as a special diet in the incidence of this disease.^(5)^ Vitamin K is one of the fat-soluble vitamins found in two forms in nature: phylloquinone (K1) and menaquinones (K2).^(6)^ Vitamin K has several effects like inhibiting vascular smooth muscle contraction, promoting the formation of blood clots, and reducing inflammation in the body.^(7)^

Various studies have examined the effect of this vitamin on cardiovascular risk factors. For example, Rahimi Sakak *et al*. study investigated the effect of vitamin K supplementation on glucose and lipid profiles in people with type 2 diabetes. According to the results of this study, supplementation with this vitamin was able to reduce fasting insulin and HbA1c, while no decrease in lipid profile was observed.^(8)^ In another study conducted by Kristensen *et al.* on vitamin K supplementation of lipid and inflammatory factors, it was found that supplementation with this vitamin worsened the lipid profile of the subjects and had no effect on other CVD risk factors.^(9)^

Nevertheless, of controversies, many studies have examined the effect of vitamin K supplementation on inflammatory factors, and lipid and glycemic profiles. Therefore, in this meta-analysis, we examined the overall effect of vitamin K supplementation therapy on inflammatory and metabolic factors affecting CVD.

## Material and methods

This study was documented according to the PRISMA [Preferred Reporting Items for Systematic Review and Meta-analysis] guidelines.^(10)^ The study protocol has been previously registered with the PROSPERO database (registration ID: CRD42023454787). Searches were performed independently by two authors, and any discrepancies between the two authors were reviewed by a third author. We conducted a pervasive systematic search in PubMed/MEDLINE, SCOPUS, Web of Science, and Embase from inception until April 2022 without using time or language restrictions. The randomized clinical trials (RCTs) that explain the effect of vitamin K supplementation on blood glucose, HbA1c, HOMA-IR, BMI, body weight (BW), WC, triglyceride (TG), LDL, HDL, total cholesterol (TC), and BP levels were selected. Also, medical subject heading (MeSH) were obtained in order to search the online databases, as follows: (‘Vitamin K’) AND (‘Glycated Haemoglobin A’ OR ‘Insulin Resistance’ OR Insulin OR Glucose OR ‘Glucose Intolerance’ OR ‘Waist Circumference’ OR ‘Body Mass Index’ OR Triglycerides OR ‘Cholesterol, HDL’ OR ‘Cholesterol, LDL’ OR ‘Blood Pressure’ OR ‘Hypertension’ OR ‘Arterial Pressure’) AND (‘Clinical Trials as Topic’ OR ‘Cross-Over Studies’ OR ‘Double-Blind Method’ OR ‘Single-Blind Method’ OR ‘Random Allocation’ OR ‘Controlled Clinical Trials as Topic’) (see Appendix 1 in Supplementary Material for search terms used across the PubMed/MEDLINE database). The reference lists of the publications retrieved and linked review studies were manually searched to identify potentially overlooked qualifying trials.

### Study selection

First, we excluded duplicate articles and then screened the title, abstract, and full text of the studies in order to detect suitable ones. Eventually, studies that have the following criteria, included: (1) Be RCTs (not animal studies, case reports, review, and meta-analysis articles, or observational), (2) Prescribing vitamin K supplementation as a pure intervention, (3) Participating in healthy or unhealthy adults (18 years old or older) and reporting anthropometric indexes (BMI, BW, WC, Weight, Height), BPs (SBP, DBP), inflammatory biomarkers (CRP), glycemic (HbA1c, FBS, Insulin, HOMA-IR), and lipid profiles (TG, LDL, HDL, TC). The duplicated data, studies with unclear information and which did not receive any feedback from the corresponding author(s) after email, non-randomized study designs, animal and observational studies, studies without a control group, and reviews were excluded. Also, the studies that reported the duration of the intervention in hours were excluded from this study.

### Data extraction

We obtained and revised the following data: author, year of publication, country, number of intervention and control groups, participation information like gender, health conditions, mean BMI and age, duration of intervention, means, and standard deviations of TG, LDL, HDL, TC, BP (systolic and diastolic), FBS, HbA1c, HOMA-IR, CRP, BW, BMI at baseline, post-treatment, and/or changes between baseline and treatment. The software WebPlot digitizer, version 4.5 2021; https://automeris.io/WebPlotDigitizer/ (accessed on 22 November 2022) was used to extract data from graphs.

### Quality assessment

As shown in Table 2, in order to assess study quality and the quality of contained RCTs was methodologically assessed using the Cochrane risk-of-bias test for randomized trials (RoB 2), version 2.^(11)^ This method assesses study quality in 7 domains: 1. Random Sequence Generation, 2. Allocation concealment, 3. Blinding of participants and personnel, 4. Blinding of outcome assessment, 5. Incomplete outcome data, 6. selective outcome reporting, 7. Other Sources of bias. As determined, among these 17 trials, we found 2 trials that had missing data^(12,13)^ and one of them was high in the overall assessment of risk of bias because it was unclear or high risk in some other item.^(14)^ Also, NutriGrade (Grading of Recommendations Assessment, Development, and Evaluation) scoring system was used to evaluate the quality of the current analysis study.^(15)^ The NutriGrade checklist is a valid 10-point scoring system that measures factors influencing study quality. This scale includes seven items (1) risk of bias, study quality, and study limitations, (2) precision, (3) heterogeneity, (4) directness, (5) publication bias, (6) funding bias, (7) study design.

### Statistical analysis

In this study, STATA version 12⋅0 (Stata Corp, College Station, TX, USA) was used for statistical analysis. Different data types were converted using a predetermined procedure to the mean and standard deviations (SDs). For example, if the data are reported as the median and 95 % confidence interval, those were converted to the mean using the appropriate formula’. For instance, in the absence of standard deviations, we calculated the change using the method below: The definition of standard deviation changes is the square root [(SD baseline 2 + sd final 2) – (2R SD baseline 2 SD final)]. In addition, according to the reference, we used a constant number of 0⋅5 to calculate *R*.^(16)^ The following formula is used to convert the standard error of the mean (sem) to standard deviation: SD is equal to sem × √*n*, where *n* is the total number of participants in each group. Also, heterogeneity was assayed using the *I*-squared (*I*^2^) statistic, and there are two conditions to realize the source of heterogeneity: if the *I*^2^ value was >50 %, or if they're in the case of inconsistency across RCTs data.^(17)^ Then, to clear the potential source of heterogeneity, we performed a subgroup analysis based on the dose of vitamin K supplementation, duration of intervention, and type of vitamin K supplementation (K1 and K2). To meta-analysis of our outcomes, we used the random-effect model. A sensitivity analysis was done to determine the contribution of each research to the overall mean difference.

## Results

**Fig. 1.**
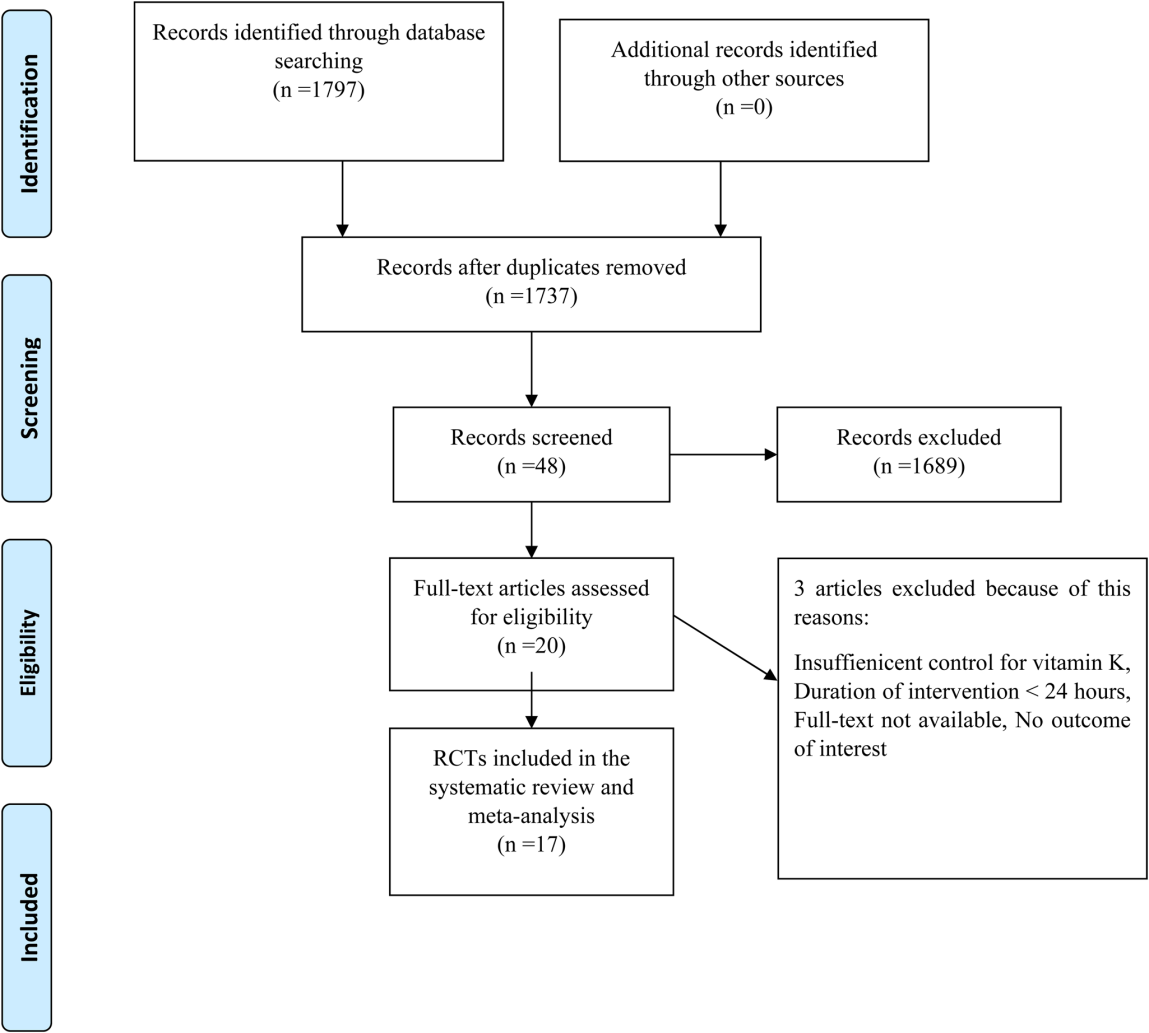
Flow chart of the study, including identification, screening, eligibility, and the final sample included.

Fig. 1 shows a flowchart of the study selection process and reasons for excluding articles. Then, 1797 publications from the aforementioned electronic databases were yielded in this figure. After excluding duplicate studies, a total of 1737 publications were remained. Then, we reviewed the title/abstract of the studies and excluded articles which did not meet the inclusion criteria. Twenty articles were retrieved during the secondary screening by full text. Of those, three studies were discarded for the different reasons. Finally, seventeen studies met the eligibility criteria and were included in the quantitative meta-analysis.

### Study characteristics

Characteristics of the pooled studies are determined in Table 1. Five studies were carried out in Iran, two studies were conducted in the USA, Denmark, the Netherlands, and Japan, one study was carried out in U.K, Greece, and Poland and finally, the country of one study was unknown. All of the articles were published between 2008 and 2021. Also, the age range of participants was 40⋅25–70⋅18 and the BMI range of them was 21⋅1–29⋅44. In some of the articles both sexes, in some of the others, only women, and just in one study just men participated. As determined, all detected studies are RCT with parallel design except one that is crossover. The duration of intervention in all RCTs ranged from 4 to 144 weeks. The dose used for supplementation in studies ranged from 0⋅09 to 400 mg/d for the intervention group and the same placebo for the control group (except for two studies that didn't mention any product for the control group and one study that didn't give anything to them). Among the total studies contained in our meta-analysis, five studies were conducted on postmenopausal women, four studies were performed on people with renal dysfunction or renal transplantation, three studies were on persons with diabetic or prediabetes problems, and every four remaining studies were conducted on a group of participants with polycystic ovary syndrome, vascular disease, rheumatoid arthritis, older healthy people, and not mentioned disease, respectively.

**Table 1.** Characteristics of eligible studies

Author (year)	Country	Clinical trial design	Population	Mean age year	Sex	Sample size Case/Placebo	Period (week)	Mean BMI	Intervention group	Control group
Sofie Hertz Rønn (2021)	Denmark	Randomized placebo-controlled trial	Postmenopausal women	68	Female	71/71	52	23⋅7	MK-7 375 μg a day	Placebo in addition to two tablets of calcium (400 mg each) and vitamin D
Sofie Hertz Rønn (2021)	Denmark	Randomized placebo-controlled trial	Postmenopausal women	68	Female	50/47	52	23⋅7	MK-7 375 μg a day	Placebo in addition to two tablets of calcium (400 mg each) and vitamin D
Fatemeh Rahimi Sakak1 (2020)	Iran	Double-blinded, placebo-controlled, randomized trial	Diabetes mellitus	58⋅5	Female/Male	32/23	12	27⋅5	180 μg vitamin K2 capsules in the form of MK-7 twice a day	Placebo capsules twice a day
Miles D. Witham (2020)	UK	Double-blind, randomized trial	Chronic kidney disease	66	Female/Male	80/79	48	N/A	400 μg oral vitamin K2 once daily	Placebo
Tarkesh (2020)	Iran	Randomized, double-blind, placebo-controlled clinical trial	Polycystic ovary syndrome	N/A	Female/Male	42/42	8	N/A	90 μg Menaquinone-7 daily	Placebo
Karamzad (2020)	Iran	randomized, double-blind, placebo-controlled clinical trial	Type 2 diabetes mellitus	N/A	Female	30/30	12	N/A	200 μg menaquinone-7 daily	Placebo
Theodora Oikonomaki (2019)	Greece	Open-labeled prospective randomized trial	ESRD on haemodialysis	70⋅18	Female/Male	22/30	52	N/A	200 μg of vitamin K2 orally (vitamin K2/MK-7, Solgar) every day	Nothing
Anthony G. Mansour (2017)	USA	Single-centre, single-arm trial	Renal transplant recipients	49⋅7	Female/Male	51	8	N/A	Menaquinone-7 360 μg once daily	N/A
Fulton (2016)	Netherlands	A double blind, randomized, placebo-controlled trial	Vascular disease	N/A	Female/Male	40/40	24	N/A	100 μg vitamin K2 daily	Placebo
Sousan Kolahi (2015)	Iran	Randomized, double-blind, placebo-controlled trial	Rheumatoid arthritis	37⋅97	Female	30/28	8	29⋅44	Vitamin K1 as phylloquinone [10 mg/d]	Placebo
H Rasekhi (2015)	Iran	Randomized, double-blinded, placebo-controlled clinical trial	Prediabetes	40⋅25	Female	39/43	4	28⋅34	1000 μg of phylloquinone daily	Placebo
Ilona Kurnatowska1 (2015)	Poland	Prospective, randomized, and double-blind study	Non-dialysed patients with chronic kidney disease, stages 3–5.	Men 60 women 56	Female/Male	28/13	36	N/A	90 μg of vitamin K2 (menaquinone-7, MK-7) plus 10 μg of cholecalciferol per day	10 μg of cholecalciferol per day
Noriko Koitaya (2013)	Japan	Randomized, double-blind, placebo-controlled trial	Healthy postmenopausal women	58/3	Female	24/24	48	N/A	1⋅5 mg MK-4	Placebo
G.W. Dalmeijer (2012)	Netherlands	Randomized, double-blind, placebo-controlled trial	N/A	N/A	Female/Male	22/20	12	N/A	180 μg/d MK-7	Placebo
G.W. Dalmeijer (2012)	Netherlands	Randomized, double-blind, placebo-controlled trial	N/A	N/A	Female/Male	18/20	12	N/A	360 μg/d MK-7	Placebo
Rajiv Kumar (2010)	USA	Clinical trial	Postmenopausal women	N/A	Female	21/21	48	N/A	1 mg phylloquinone daily	N/A
Noriko Koitaya (2008)	Japan	Randomized double-blind	Postmenopausal women	59⋅8	Female	N/A	4	21⋅1	1/5 mg MK-4 a day	Placebo
Makiko Yoshida (2008)	N/A	Randomized, double-blind, controlled trial	Older nondiabetic	N/A	Male	80/62	144	N/A	500 μg/d phylloquinone	Placebo
Makiko Yoshida (2008)	N/A	Randomized, double-blind, controlled trial	Older nondiabetic	N/A	Female	104/109	144	N/A	500 μg/d phylloquinone	Placebo
Mette Kristensen (2008)	Denmark	A randomized, double-blinded crossover design	Postmenopausal women	62⋅5	Female	31/31	6	26⋅1	500 μg of phylloquinone	Placebo

ESRD, end stage of renal disease; MK, menaquinone; N/A, not available

Table 2 represents the results of the quality assessment of accepted studies. Also, after evaluating the quality of the present meta-analysis based on the NutriGrade score system, a score of 8⋅2 (very good quality) was calculated.

**Table 2.** Risk of bias assessment according to the Cochrane collaboration's risk of the bias assessment tool

Study, year (reference)	Random sequence generation	Allocation concealment	Blinding of participants and personnel	Blinding of outcome assessment	Incomplete outcome data	Selective reporting	Overall assessment of risk of bias
Sofie Hertz Rønn (2021)	L	U	L	L	L	L	L
Fatemeh Rahimi Sakak1 (2020)	L	L	L	L	L	L	L
Miles D. Witham (2020)	L	L	L	L	H	L	L
Tarkesh (2020)	L	L	L	L	L	L	L
Karamzad (2020)	L	L	L	L	L	L	L
Theodora Oikonomaki (2019)	U	U	H	H	L	L	H
Anthony G. Mansour (2017)	U	U	U	U	L	L	U
R.L. FULTON (2016)	L	L	L	L	H	L	L
Sousan Kolahi (2015)	U	U	L	U	L	L	U
H Rasekhi (2015)	U	L	L	L	L	L	L
Ilona Kurnatowska1 (2015)	L	L	L	L	L	L	L
Noriko Koitaya (2013)	L	U	L	L	L	L	L
G.W. Dalmeijer (2012)	L	L	L	L	L	L	L
Rajiv Kumar (2010)	U	U	L	L	L	L	U
Noriko Koitaya (2008)	L	U	L	L	L	L	L
Makiko Yoshida (2008)	U	U	L	L	L	L	U
Mette Kristensen (2008)	U	U	L	L	L	L	U

L, low; H, high; U, unclear.

### Meta-analysis

#### The effect of vitamin K supplementation on glucose, insulin, HbA1c, and HOMA-IR

**Fig. 2.**
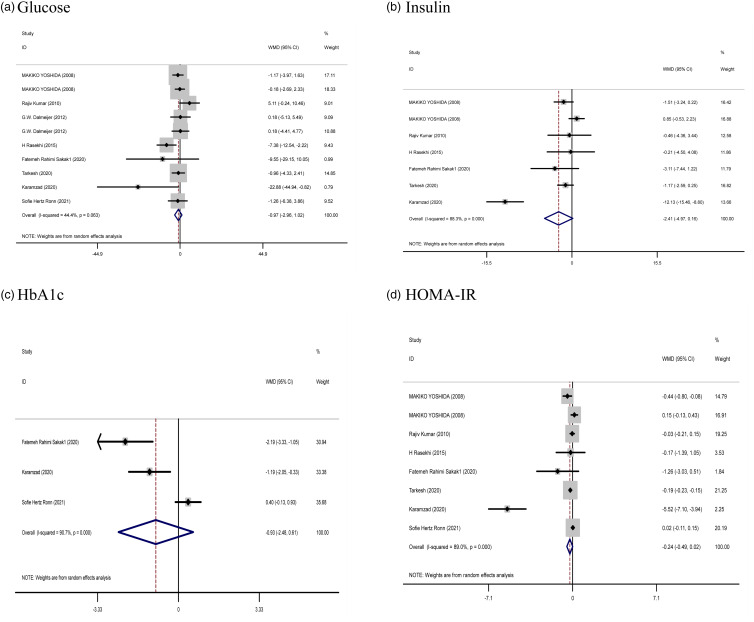
Forest plots from the meta-analysis of clinical trials investigating the effects of vitamin K supplementation on (a) glucose, (b) insulin, (c) HbA1c, and (d) HOMA-IR. WMD, weighted mean.

The pooled results obtained from seven articles showed that HOMA-IR was significantly reduced following vitamin K supplementation compared (WMD: −0⋅24, 95 % confidence interval (CI): −0⋅49, −0⋅02, *P* = 0⋅047) with placebo. However, with the use of the random effects model, the pooled results (eight studies for glucose, six articles for insulin, and three studies for HbA1c) indicated that vitamin K supplementation had no significant effect on glucose (WMD: −0⋅97 mg/dl, 95 % CI: −2⋅96, 1⋅02, *P* = 0⋅339), insulin (WMD: −2⋅41 μU/ml, 95 % CI: −4⋅97, 0⋅16, *P* = 0⋅667), and HbA1c (WMD: −0⋅93, 95 % CI: −2⋅48, 0⋅61, *P* = 0⋅237) compared with control. A high heterogeneity was shown in the trials for HbA1c (Cochran *Q* test, *P* < 0⋅001, *I*^2^ = 90⋅7 %) and HOMA-IR (Cochran *Q* test, *P* < 0⋅001, *I*^2^ = 88⋅3 %), But high heterogeneity was reported for insulin (Cochran *Q* test, *P* < 0⋅001, *I*^2^ = 88⋅3 %). However, no significant heterogeneity was reported for glucose (Cochran *Q* test, *P* = 0⋅063, *I*^2^ = 44⋅4 %) (Fig. 2).

Subgroup findings also did not show a greater potential effect of vitamin K consumption on all factors of glucose metabolism. However, subgroup analysis showed a significant effect of vitamin K2 supplementation compared to vitamin K1 supplementation on HOMA-IR. However, no significant effect was observed on other variables (Supplementary Table 1).

#### The effect of vitamin K supplementation on body composition

**Fig. 3.**
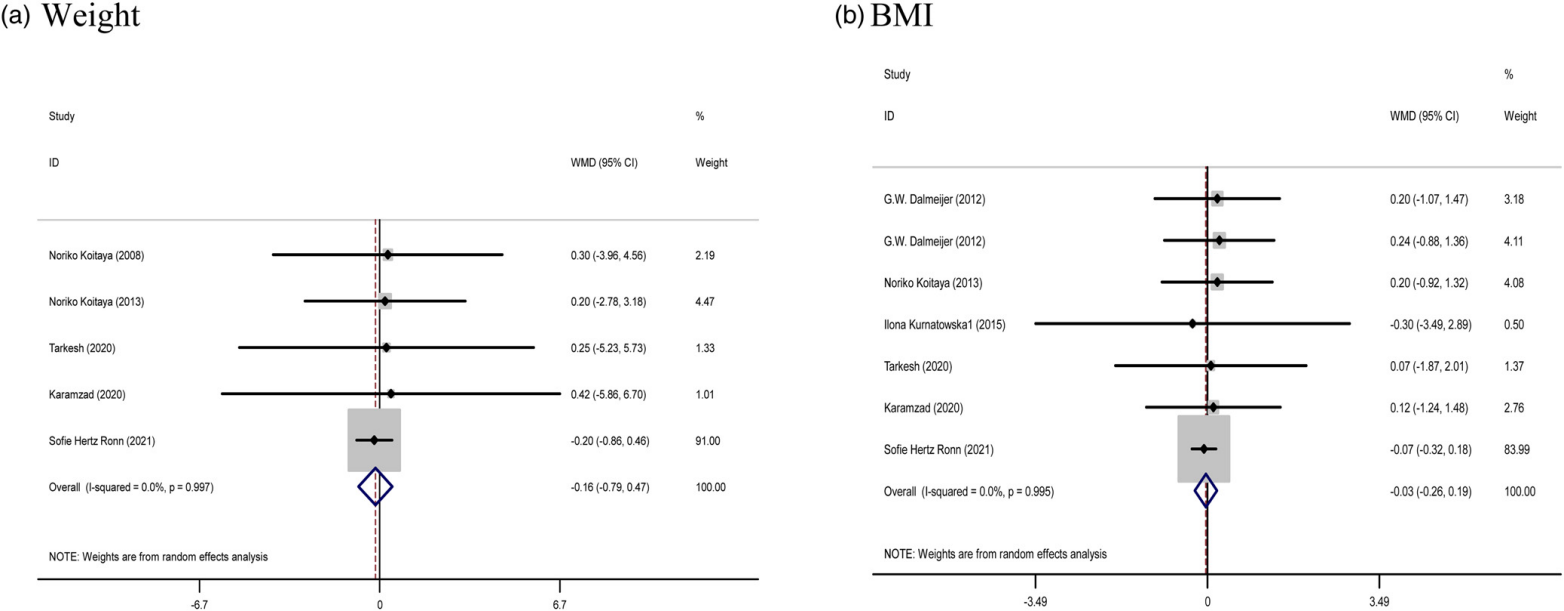
Forest plots from the meta-analysis of clinical trials investigating the effects of vitamin K supplementation on (a) weight and (b) BMI. WMD, weighted mean.

The effect of Vitamin K supplementation on body composition (the pooled results obtained from five and six studies were used for weight and BMI, respectively) showed no significant difference in the weight (WMD: −0⋅16 kg, 95 % CI: −0⋅79, 0⋅47, *P* = 0⋅621) and BMI (WMD: −0⋅03 %, 95 % CI: −0⋅26, 0⋅19, *P* = 0⋅784) compared with control. There was no evidence of significant between-study heterogeneity (Cochran *Q* test, *P* = 0⋅997, *I*^2^ = 0⋅0 % for weight; Cochran *Q* test, *P* = 0⋅995, *I*^2^ = 0⋅0 % for BMI), in all meta-analyses (Fig. 3).

Subgroup findings also did not show a greater potential effect of vitamin K consumption on BMI and weight (Supplementary Table 1).

#### The effect of vitamin K supplementation on lipid profile and CRP

**Fig. 4.**
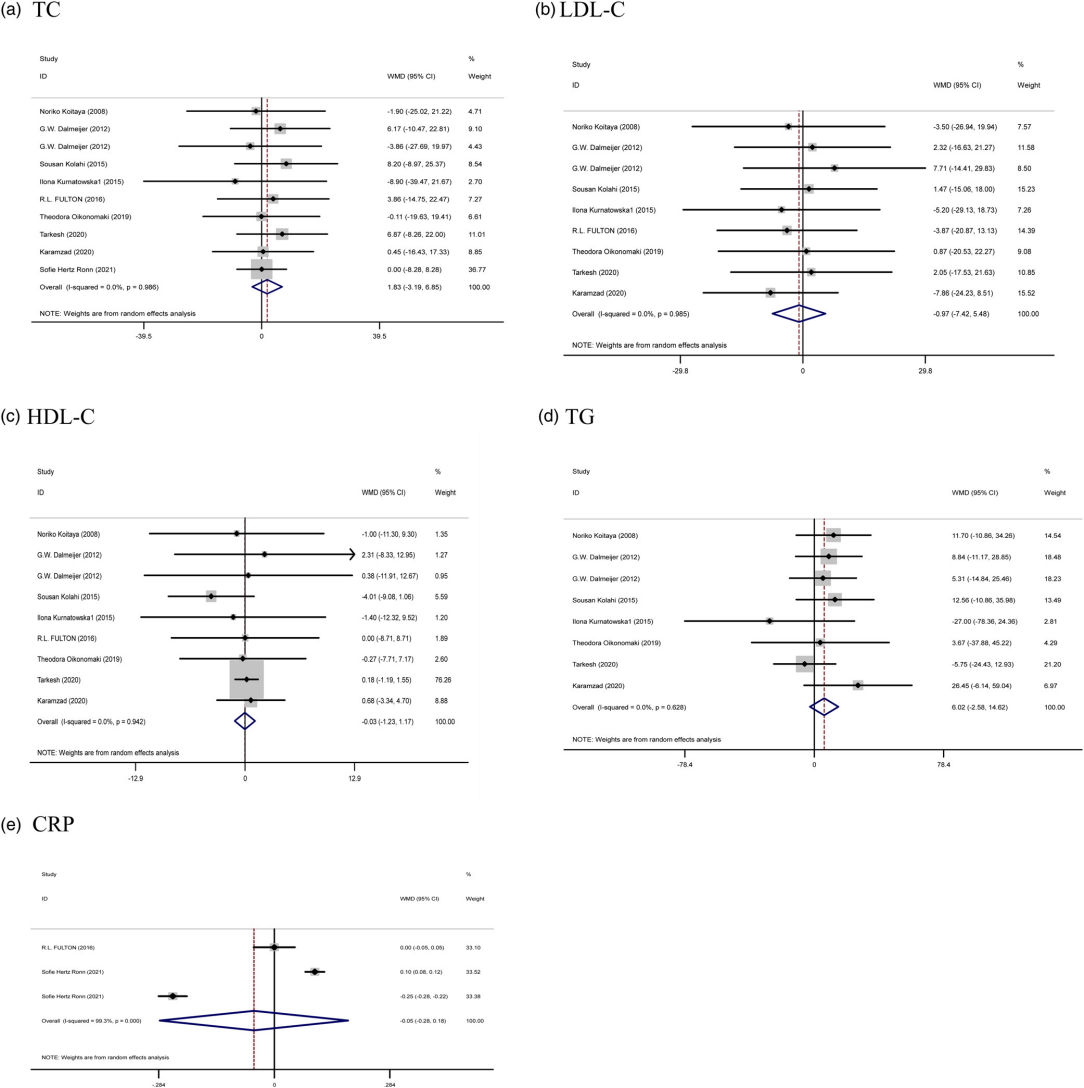
Forest plots from the meta-analysis of clinical trials investigating the effects of vitamin K supplementation on (a) TC, (b) LDL, (c) HDL, (d) TG, and (e) CRP. WMD, weighted mean.

The results of the combined data (nine articles for TC, eight articles for LDL and HDL, seven studies for TG, as well as two articles for CRP) did not show a significant effectiveness of the study on serum TC (WMD: 1⋅83 mg/dl, 95 % CI: −3⋅19, 6⋅85, *P* = 0⋅475) and TG (WMD: 6⋅02 mg/dl, 95 % CI: −2⋅58, 14⋅62, *P* = 0⋅170), LDL (WMD: −0⋅97 mg/dl, 95 % CI: −7⋅42, 5⋅48, *P* = 0⋅768), HDL (WMD: −0⋅03 mg/dl, 95 % CI: −1⋅23, 1⋅17, *P* = 0⋅960), and CRP (WMD: −0⋅05 mg/l, 95 % CI: −0⋅28, 0⋅18, *P* = 0⋅673) following vitamin K supplementation. Furthermore, no significant heterogeneity was observed between these trials for TC (Cochran *Q* test, *P* = 0⋅986, *I*^2^ = 0⋅0 %), TG (Cochran *Q* test, *P* = 0⋅628, *I*^2^ = 0⋅0 %), HDL (Cochran *Q* test, *P* = 0⋅942, *I*^2^ = 0⋅0 %), and LDL-C level (Cochran *Q* test, *P* = 0⋅985, *I*^2^ = 0⋅0 %) (Fig. 4).

Subgroup analysis based on dose and duration of intervention did not show a significant effect on any of the lipids and CRP (Supplementary Table 1).

#### Effect of vitamin K supplementation on BP

**Fig. 5.**
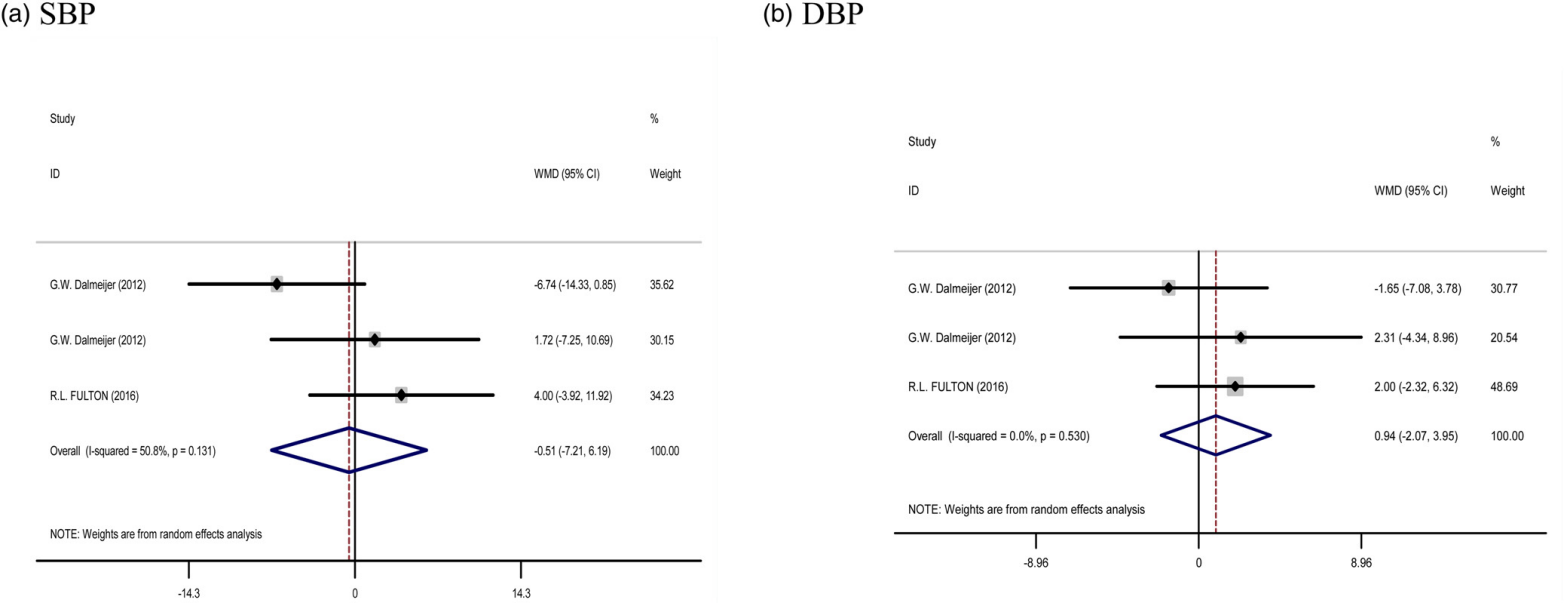
Forest plots from the meta-analysis of clinical trials investigating the effects of vitamin K supplementation on (a) SBP and (b) DBP. WMD, weighted mean.

Five treatment arms provided information for BP as an outcome measure. Pooled results from the random effects model indicated that SBP (WMD: −0⋅51 mm/Hg, 95 % CI: −7⋅21, 6⋅19, *P* = 0⋅881) and DBP (WMD: 0⋅94 mm/Hg, 95 % CI: −2⋅07, 3⋅95, *P* = 0⋅540) levels following Vitamin K supplementation did not change significantly, with a low heterogeneity being noted among the studies for SBP (*I*^2^ = 50⋅8 %, *P* = 0⋅131) and DBP (*I*^2^ = 0⋅0 %, *P* = 0⋅530) (Fig. 5).

### Sensitivity analysis

We removed each study from the analysis, step by step to discover the impact of each single trial on the pooled effect size for weight, BMI, TC, LDL, and HDL cholesterol and TG, glucose, insulin, HbA1c, HOMA-IR, SBP, DBP, and CRP level. The leave-one-out sensitivity analysis showed the robustness of the findings (Supplementary Figures 1–4).

## Discussion

This meta-analysis and systematic review of 17 RCTs was performed to reveal the effect of vitamin K supplementation on weight, BMI, BP, CRP, glycemic, and lipid profile. Based on our analysis, we found no significant effect of vitamin K supplementation on CVD risk factors except HOMA-IR compared with the placebo group. Also, subgroup analysis according to the duration of trials or dosage of vitamin k didn't show any effect on detected variables. It means long supplementation with vitamin K or even a high intake of it (≥500 μg) doesn't help to improve glucose and lipid-related factors or reduce weight, and CRP levels.

Like ours, Shahdadian *et al.*^(18)^ reported no significant effect on vitamin K supplementation and the glycemic profile in their meta-analysis. Also, Suksomboon *et al.*^(19)^ show in their review that supplementation with vitamin K has no effect on HOMA-IR, fasting glucose and insulin, IL-6, and even CRP. Besides, a review that Varsamis *et al.* published,^(20)^ represents that vitamin K status is related to better glucose and lipid metabolism and it can be an acceptable strategy to prevent or even treat type 2 diabetes mellitus. The difference between the results of our study and this review may be due to the difference in the inclusion criteria. Our study is a meta-analysis of RCTs and this study is a review of original studies, even *in vitro* articles. As a result, the compared populations are different.

Despite our findings, there are some mechanisms that describe the role of Vitamin K in improving glycemic and lipid profiles. This vitamin can do its role by modulating inflammatory factors and acting on osteocalcin (OC), growth-arrest-specific gene 6 (GAS6), prothrombin, and protein S through its effect on the pancreas and liver. For example, osteocalcin controls the glucose profile of those taking vitamin K by affecting the proliferation of pancreatic beta cells and increasing insulin secretion, as well as increasing the production of adiponectin in fat cells.^(21,22)^ Studies on mice have shown decreased levels of under- γ-carboxylated osteocalcin (ucOC) are associated with impaired blood glucose balance, and if injected into mice, glucose homeostasis will be established and insulin gene expression will be increased. Matrix Gla protein (MGP) is a member of the family of Gla-containing proteins dependent on vitamin K2, which seems to be effective in the processes of glucose metabolism and even inflammatory processes.^(23)^ However, such an effect has not been observed in human studies,^(24)^ which could be a reason for the ineffectiveness of vitamin K supplementation on most glycemic indexes. Also, Ronn *et al.*, suggest that the glucose tolerance test is better to investigate the β-cell function and postprandial glucose metabolism,^(25)^ which isn't measured in most studies. In addition, vitamin K can indirectly inhibit lipid production by suppressing gluconeogenesis. Also, this vitamin can regulate insulin and glucose by lowering TC and LDL while increasing HDL.^(26)^

Furthermore, some studies said that the mechanism of action of vitamin K on changes in lipid profile, especially cholesterol,^(27)^ and consequently its effect on cardiovascular health is not clear,^(28)^ but they hypothesize that supplementation with this vitamin in addition to vitamin D, because of their synergic effects, can influence cardiovascular health by affecting on bone health.^(29)^ It is stated that some proteins necessary for the health of this system are activated by the process of vitamin K-dependent gamma-carboxylation. For example, the carboxylated MGP and osteocalcin can prevent the calcification of vascular tissue. They affect by limiting the incorporation of Calcium in soft tissue and controlling bone mineralization, respectively. On the other hand, it has been demonstrated that vitamin D increases MGP mRNA expression in humans, and also it has been understood that vitamin K through 1,25 dihydroxyvitamin D can stimulate the accumulation and mineralization of osteocalcin.^(28,29)^ So, this synergic effect of these vitamins accounts for preventing arterial stiffness and it can be the reason why our result didn't show any positive effect on vitamin K supplementation and changing SBP or DBP. Maybe analysing the synergic effect of vitamin K and D could result in the same consequence. Furthermore, it has been understood that TG-rich lipoproteins are a carrier for vitamin K, so it can reveal the positive relationship between this vitamin and TG level.^(27)^ Anyway, we didn't find any remarkable relevance between lipid status and vitamin K supplementation; maybe it would be better if we analysed the relationship between lipoproteins and supplementation of this vitamin.^(28)^

Vitamin K reduces lipogenesis by inhibiting pro-inflammatory cytokines as well as nuclear factor kappa-light-chain-enhancer of activated B cells (NF-kB)and thus inhibiting peroxisome proliferator-activated receptor gamma (PPAR-γ).^(26)^ So, it seems that CRP is not a suitable inflammatory biomarker to show the effect of vitamin K supplementation because it's not one of the special inflammatory markers in vitamin K's metabolism pathway and it can be changed in every stressful condition.

In the change of BW, no significant results were observed. Actually, as Knapen *et al.*^(30)^ revealed, vitamin K seems to maintain weight by inhibiting adipogenesis and some cellular processes independent of gamma-carboxylation, and it could be a reason for our result.

The strength of our meta-analysis is using subgroup analysis to find the source of heterogeneity if there was. Also, we include RCT studies which used didn't combine any other supplement with vitamin K. In addition, we used the GRADE assessment tool to rate the certainty of the evidence for all of the outcomes. Besides, our study has some limitations too: First, remarkable heterogeneity was found for HbAlc, HOMA-IR, and insulin which can be caused by different populations included in the study as well as some individual characteristics such as genetic differences, age, physical activity, and weight. Also, different diseases and different types of participants influenced the result of every RCTs and the overall result of our meta-analysis. In addition, this study was conducted with the aim of investigating the effect of vitamin K alone on the mentioned factors, while some studies had examined the effect of this vitamin at the same time as receiving vitamin D by the intervention and placebo groups, which complicated the interpretation of the results. In addition, the lack of subgroup analysis based on study populations due to the small number of studies in each subgroup was another limitation of our study.

## Conclusion

In conclusion, we demonstrate from these seventeen RCTs that vitamin K supplementation has no significant effect on changing weight, reducing CRP, controlling blood pressure, or even factors that belong to glycemic and lipid status, such as glucose, HbA1c, insulin, TC, TG, LDL, HDL except HOMA-IR.

## Supporting information

Zhao et al. supplementary material 1 Zhao et al. supplementary material

Zhao et al. supplementary material 2 Zhao et al. supplementary material

Zhao et al. supplementary material 3 Zhao et al. supplementary material

## Supplementary material

The supplementary material for this article can be found at https://doi.org/10.1017/jns.2023.106.
