# Supplementary material for: The effect of vitamin K supplementation on cardiovascular risk factors: a systematic review and meta-analysis
Source: J Nutr Sci. 2024 Jan 11;13:e3. doi: 10.1017/jns.2023.106 (PMC10808880; doi:10.1017/jns.2023.106)
Supplement: Zhao et al. supplementary material 1 — Zhao et al. supplementary material [file S2048679023001064sup001.docx]

***Appendix 1: Search strategies including the key terms and the queries for PubMed database***

| ***Search strategies*** |
| --- |
| ("Vitamin K" [Mesh] OR "Vitamin K"[ All Fields] OR "Vitamin K 3"[ Mesh] OR “2-Methylnaphthoquinone”[ All Fields] OR “Menadione” [All Fields] OR “Vicasol” [All Fields] OR “Vikasol”[ All Fields] OR “Vitamin K 2”[ Mesh] OR “Menaquinone” [All Fields] OR “menadiol” [All Fields] OR “menatetrenone” [All Fields] OR “‘phytonadione” [ All Fields] OR “methylphytyl” [All Fields] OR “phylloquinone” [All Fields] OR “phytomenadione” [ All Fields] OR “Mephyton” [All Fields] OR “Konakion” [ All Fields]) AND ("Glycated Hemoglobin A"[Mesh] OR "Glycated Hemoglobin A"[ All Fields]OR HbA1c[All Fields] OR "Insulin Resistance"[ All Fields] OR "Insulin Resistance"[MeSH] OR Insulin[All Fields] OR Insulin[Mesh] OR Glucose[All Fields] OR "Glucose Intolerance"[ All Fields] OR Glucose[Mesh] OR "Glucose Intolerance"[Mesh] OR "Waist Circumference"[ All Fields] OR "Waist Circumference"[Mesh] OR "Body Mass Index"[ All Fields] OR "Body Mass Index"[Mesh] OR BMI[All Fields] OR Triglycerides[All Fields] OR Triglycerides[Mesh] OR "Cholesterol, HDL"[Mesh] OR "HDL"[ All Fields] OR "Cholesterol, LDL"[Mesh] OR LDL[All Fields] OR "High-density lipoprotein"[ All Fields] OR "Low-density lipoprotein"[ All Fields] OR "Blood Pressure"[Mesh] OR "Blood Pressure"[ All Fields] OR "Arterial Pressure"[ All Fields] OR "Hypertension"[Mesh] OR "Arterial Pressure"[Mesh] OR "Hypertension"[ All Fields] OR SBP[All Fields] OR DBP[All Fields] OR "total cholesterol" [All Fields]) AND ("Clinical Trials as Topic"[Mesh] OR "Cross-Over Studies"[Mesh] OR "Double-Blind Method"[Mesh] OR "Single-Blind Method"[Mesh] OR "Random Allocation"[Mesh] OR RCT[All Fields] OR "Clinical Trial" [Publication Type] OR "Controlled Clinical Trials as Topic"[Mesh] OR "Intervention Studies"[ All Fields] OR intervention[All Fields] OR Trial[All Fields] OR "controlled trial"[ All Fields] OR randomized[All Fields] OR randomised[All Fields] OR random[All Fields] OR randomly[All Fields] OR placebo[All Fields] OR assignment[All Fields]) |
