# Supplementary material for: The effect of vitamin K supplementation on cardiovascular risk factors: a systematic review and meta-analysis
Source: J Nutr Sci. 2024 Jan 11;13:e3. doi: 10.1017/jns.2023.106 (PMC10808880; doi:10.1017/jns.2023.106)
Supplement: Zhao et al. supplementary material 2 — Zhao et al. supplementary material [file S2048679023001064sup002.docx]

| a) glucose   | b)insulin   |
| --- | --- |
| c)HbA1c   | d) HOMA-IR   |
| **Supplementary figures 1.** Sensitivity analysis of the weighted mean difference (WMD) for a) glucose, b) insulin and c) HbA1c, d) HOMA-IR | |

| a)weight   | b)BMI   |
| --- | --- |
| **Supplementary figures 2.** Sensitivity analysis of the weighted mean difference (WMD) for a) weight, b) BMI | |

| a)cholesterol   | b)LDL   |
| --- | --- |
| c)HDL   | d)TG   |
| e)CRP   |  |
| **Supplementary figures 3.** Sensitivity analysis of the weighted mean difference (WMD) for a) cholesterol, b) LDL, c) HDL, d) TG e) CRP | |

| a) SBP   | b)DBP   |
| --- | --- |
| **Supplementary figures 4.** Sensitivity analysis of the weighted mean difference (WMD) for a) SBP, b) DBP | |
