# Supplementary material for: The effect of vitamin K supplementation on cardiovascular risk factors: a systematic review and meta-analysis
Source: J Nutr Sci. 2024 Jan 11;13:e3. doi: 10.1017/jns.2023.106 (PMC10808880; doi:10.1017/jns.2023.106)
Supplement: Zhao et al. supplementary material 3 — Zhao et al. supplementary material [file S2048679023001064sup003.docx]

**Supplementary Table 1-** Meta-analyses showing the effect of vitamin K on several subgroups (all analyses were conducted using random effects model).

|  | | | | | | | | | **Heterogeneity** | |  |
| --- | --- | --- | --- | --- | --- | --- | --- | --- | --- | --- | --- |
|  | | | | | | | | **WMD^1^ (95%CI)** | ***I^2^* (%)** | ***P* within group** | |
| **Fasting glucose (mg/dl)** | | | | | | | |  |  |  | |
| ***Duration*** | *< 12 weeks* | | | | | | | **-3.86 -10.12 2.40** | ***76.0*** | ***0.041*** | |
|  | *≥ 12 weeks* | | | | | | | **-0.17 -2.10 1.78** | ***25.5*** | ***0.226*** | |
| ***Dosage*** | *< 500 µg* | | | | | | | **-0.89 -3.05 1.28** | ***0.0*** | ***0.421*** | |
|  | *≥ 500 µg* | | | | | | | **-0.89 -4.44 2.65** | ***73.3*** | ***0.011*** | |
| **Type of intervention** | *K1* | | | | | | | **-0.89 -4.44 2.65** | ***73.3*** | ***0.011*** | |
|  | *K2* | | | | | | | **-0.89 -3.05 1.28** | ***0.0*** | ***0.421*** | |
| **Insulin (μU/mL)** | | | | | | | |  |  |  | |
| ***Duration*** | *< 12 weeks* | | | | | | | **-1.07 -2.43 0.28** | ***0.0*** | ***0.677*** | |
|  | *≥ 12 weeks* | | | | | | | **-3.16 -7.08 0.77** | ***92.1*** | ***<0.001*** | |
| ***Dosage*** | *< 500 µg* | | | | | | | **-5.42 -12.49 1.65** | ***94.3*** | ***<0.001*** | |
|  | *≥ 500 µg* | | | | | | | **-0.23 -1.62 1.16** | ***32.1*** | ***0.220*** | |
| **Type of intervention** | *K1* | | | | | | | **-0.23 -1.62 1.16** | ***32.1*** | ***0.220*** | |
|  | *K2* | | | | | | | **-5.42 -12.49 1.65** | ***94.3*** | ***<0.001*** | |
| **HOMA-IR** | | | | | | | |  |  |  | |
| ***Duration*** | *< 12 weeks* | | | | | | | **-0.19 -0.23 -0.15** | ***0.0*** | ***0.974*** | |
|  | *≥ 12 weeks* | | | | | | | **-0.43 -0.85 -0.00** | ***91.0*** | ***<0.001*** | |
| ***Dosage*** | *< 500 µg* | | | | | | | **-0.55 -1.02 -0.09** | ***94.5*** | ***<0.001*** | |
|  | *≥ 500 µg* | | | | | | | **-0.08 -0.33 0.17** | ***53.9*** | ***0.089*** | |
| **Type of intervention** | *K1* | | | | | | | **-0.08 -0.33 0.17** | ***53.9*** | ***0.089*** | |
|  | *K2* | | | | | | | **-0.56 -1.02 -0.09** | ***94.5*** | ***<0.001*** | |
| **Weight (kg)** | | | | | | | |  |  |  | |
| ***Duration*** | | *< 12 weeks* | | | | | | **0.28 -3.08 3.64** | ***0.0*** | ***0.989*** | |
|  |  | *≥ 12 weeks* | | | | | | **-0.17 -0.82 0.47** | ***0.0*** | ***0.951*** | |
| ***Dosage*** | | *< 500 µg* | | | | | | **-0.19 -0.84 -0.47** | ***0.0*** | ***0.970*** | |
|  |  | *≥ 500 µg* | | | | | | **0.23 -2.21 2.68** | ***0.0*** | ***0.970*** | |
| **Body mass index (BMI) (kg/m^2^)** | | | | | | | |  |  |  | |
| ***Duration*** | | | *< 12 weeks* | | | | | **0.07 -1.87 2.01** | ***-*** | ***-*** | |
|  |  |  | *≥ 12 weeks* | | | | | **-0.03 -0.26 0.20** | ***0.0*** | ***0.984*** | |
| ***Dosage*** | | | *< 500 µg* | | | | | **-0.04 -0.27 0.19** | ***0.0*** | ***0.991*** | |
|  |  |  | *≥ 500 µg* | | | | | **0.20 -0.92 1.32** | ***-*** | ***-*** | |
| **Total cholesterol (mg/ dl)** | | | | | | | |  |  |  | |
| ***Duration*** | | | | *< 12 weeks* | | | | **5.63 -4.55 15.82** | ***0.0*** | ***0.771*** | |
|  |  |  |  | *≥ 12 weeks* | | | | **0.61 -5.16 6.38** | ***0.0*** | ***0.982*** | |
| ***Dosage*** | | | | *< 500 µg* | | | | **1.41 -3.98 6.80** | ***0.0*** | ***0.977*** | |
|  |  |  |  | *≥ 500 µg* | | | | **4.61 -9.18 18.39** | ***0.0*** | ***0.492*** | |
| **Type of intervention** | | | | *K1* | | | | **8.20 -8.97 25.37** | ***-*** | ***-*** | |
|  |  |  |  | *K2* | | | | **1.24 -4.01 6.48** | ***0.0*** | ***0.988*** | |
| **LDL cholesterol(mg/ dl)** | | | | | | | |  |  |  | |
| ***Duration*** | | | | | *< 12 weeks* | | | **0.54 -10.58 11.66** | ***0.0*** | ***0.928*** | |
|  |  |  |  |  | *≥ 12 weeks* | | | **-1.74 -9.66 6.18** | ***0.0*** | ***0.900*** | |
| ***Dosage*** | | | | | *< 500 µg* | | | **-1.20 -8.55 6.14** | ***0.0*** | ***0.942*** | |
|  |  |  |  |  | *≥ 500 µg* | | | **-0.18 -13.69 13.33** | ***0.0*** | ***0.734*** | |
| **Type of intervention** | | | | | *K1* | | | **1.47 -15.06 18.00** | ***-*** | ***-*** | |
|  |  |  |  |  | *K2* | | | **-1.44 -8.42 5.60** | ***0.0*** | ***0.972*** | |
| **HDL cholesterol(mg/ dl)** | | | | | | | |  |  |  | |
| ***Duration*** | | | | | | *< 12 weeks* | | **-0.63 -2.97 1.72** | ***19.1*** | ***0.290*** | |
|  |  |  |  |  |  | *≥ 12 weeks* | | **0.41 -2.51 3.34** | ***0.0*** | ***0.998*** | |
| ***Dosage*** | | | | | | *< 500 µg* | | **0.22 -1.02 1.46** | ***0.0*** | ***0.999*** | |
|  |  |  |  |  |  | *≥ 500 µg* | | **-3.42 -7.97 1.13** | ***0. 0*** | ***0.60*** | |
| **Type of intervention** | | | | | | *K1* | | **-4.01 -9.08 1.06** | ***-*** | ***-*** | |
|  |  |  |  |  |  | *K2* | | **0.20 -1.03 1.44** | ***0.0*** | ***1.00*** | |
| **Triglycerides (mg/ dl)** | | | | | | | |  |  |  | |
| ***Duration*** | | | | | | | *< 12 weeks* | **2.76 -10.11 15.63** | ***10.8*** | ***0.339*** | |
|  |  |  |  |  |  |  | *≥ 12 weeks* | **9.59 -2.83 22.02** | ***0.0*** | ***0.733*** | |
| ***Dosage*** | | | | | | | *< 500 µg* | **3.65 -6.49 13.79** | ***0.0*** | ***0.479*** | |
|  |  |  |  |  |  |  | *≥ 500 µg* | **12.11 -4.13 28.36** | ***0.0*** | ***0.959*** | |
| **Type of intervention** | | | | | | | *K1* | **12.58 -10.86 35.98** | ***-*** | ***-*** | |
|  |  |  |  |  |  |  | *K2* | **5.00 -4.25 14.25** | ***0.0*** | ***0.555*** | |
